# Redox homeostasis dysregulation in noise-induced hearing loss: oxidative stress and antioxidant treatment

**DOI:** 10.1186/s40463-023-00686-x

**Published:** 2023-12-11

**Authors:** Yuhang Zhou, Chaoyou Fang, Ling Yuan, Mengchen Guo, Xinyi Xu, Anwen Shao, Anke Zhang, Danyang Zhou

**Affiliations:** 1 Health Management Center, Tongde Hospital of Zhejiang Province, Hangzhou, China; 2 The First Clinical Medical College, Heilongjiang University of Chinese Medicine, Harbin, China; 3 Department of Neurosurgery, Shanghai General Hospital, School of Medicine, Shanghai Jiao Tong University, Shanghai, China; 4 Department of Dermatology, Tongji Hospital, School of Medicine, Tongji University, Shanghai, China; 5 School of Medicine, Ningbo University, Ningbo, China; 6 Department of Neurosurgery, The Second Affiliated Hospital, School of Medicine, Zhejiang University, Hangzhou, China

**Keywords:** Noise-induced hearing loss, Oxidative stress, Antioxidant treatment

## Abstract

Noise exposure is an important cause of acquired hearing loss. Studies have found that noise exposure causes dysregulated redox homeostasis in cochlear tissue, which has been recognized as a signature feature of hearing loss. Oxidative stress plays a pivotal role in many diseases via very complex and diverse mechanisms and targets. Reactive oxygen species are products of oxidative stress that exert toxic effects on a variety of physiological activities and are considered significant in noise-induced hearing loss (NIHL). Endogenous cellular antioxidants can directly or indirectly counteract oxidative stress and regulate intracellular redox homeostasis, and exogenous antioxidants can complement and enhance this effect. Therefore, antioxidant therapy is considered a promising direction for NIHL treatment. However, drug experiments have been limited to animal models of NIHL, and these experiments and related observations are difficult to translate in humans; therefore, the mechanisms and true effects of these drugs need to be further analyzed. This review outlines the effects of oxidative stress in NIHL and discusses the main mechanisms and strategies of antioxidant treatment for NIHL.

## Introduction

Among the numerous causes of hearing loss, noise exposure is an important contributor, particularly in noise-induced hearing loss (NIHL). Exposure to excessive noise leads to hair cell (HC) loss, auditory nerve degeneration and synaptic reduction at the microscale, and these effects manifest as loss of auditory sensitivity and a temporary or permanent hearing threshold shift [1]. Mild or moderate noise can lead to a temporary hearing threshold shift (TTS), at which time HC damage and auditory nerve fiber (ANF) degeneration are reversible [2–4]. However, severe or long-term noise exposure can lead to cochlear HC necrosis and apoptosis, resulting in damage that cannot be restored to the pre-trauma level, a condition called permanent threshold shift (PTS) [5, 6]. According to the World Health Organization, the prevalence of NIHL among adults worldwide is 16%, with significant regional differences [7]. In addition to hearing-related symptoms, patients with NIHL often present with headaches, hypertension, tinnitus, dizziness and other symptoms, which have a serious impact on their communication and quality of life [3, 8].

The main causes of NIHL include mechanical damage from noise itself and toxic damage from oxidative stress. At the cellular level, excessive noise extensively destroys synapses between ANFs [9], which leads to the loss of the peripheral axons of bipolar sensory neurons and eventually the degeneration of cell bodies in the spiral ganglia [9, 10]. Studies have shown that outer hair cells (OHCs) are the main targets of noise damage, and OHC loss in the basal (high frequency) cochlea is exacerbated [11]. In addition to mechanical damage, noise exerts a damaging effect on the stria vascularis, which leads to a decrease in blood flow and, together with the former, leads to a threshold elevation [10, 12]. Moreover, several pathways are activated in the pathogenesis of NIHL. Autophagy is a recently discovered highly conserved eukaryotic cell cycle process that helps maintain internal homeostasis by degrading organelles, proteins, and macromolecules and recycling materials [13–15]. Autophagy is involved in the development of NIHL via β-alanine metabolism and arginine and proline metabolism and exerts an inhibitory effect on NIHL [16–18]. As the main markers of autophagy, the mRNA expression levels of lc3b and Beclin1 increased significantly after noise exposure and were localized to cochlear spiral ganglion neurons (SGNs), as determined by immunofluorescence assay [16]. For verification, researchers used the direct autophagy inhibitor 3-MA and the mTOR signaling agonist RAP [19]. The results showed that the inhibition of autophagy significantly aggravated the noise-induced oxidative imbalance, which ultimately manifested as NIHL [20]. Some inflammatory mediators are involved in mediating NIHL. The cochlea is considered an immune-exempt organ because of its hemolymph barrier [21], but it has been proven that noise exposure can induce HC ischemia [22–24]. In addition, as a key nuclear factor, NF-κB is activated and translocated under the action of a variety of damage-associated molecular patterns (DAMPs), toll-like receptors (TLRs) and receptors for advanced glycation end products (RAGEs) [1], and noise-induced damage activates the NF-κB signaling cascade [25–27]. As an example, researchers have found that NF-κB is activated 2–6 h after PTS-inducing noise [28] and thus causes HC injury and NIHL [29]. This evidence shows that oxidative stress plays an extremely important role in NIHL.

Oxidative stress is usually defined as an imbalance between oxidant and antioxidant levels, and this definition has been refined to include the abrogation of redox signaling and regulatory controls [30, 31]. Currently, oxidative stress is the focus of research in cancer, inflammation and other diseases [32–34], and these studies have promoted the progressive understanding of neurodegenerative diseases to a large extent [35–37]. For NIHL, research on oxidative stress is not abundant, but oxidative stress has been shown to induce and aggravate the progression of NIHL [38, 39]. For example, researchers have used superoxide and lipid peroxidation markers to demonstrate the level of oxidative damage in HCs and SGNs. The results showed that HCs and SGNs in the most severely damaged medial-basal area of the noise-exposed rat cochlea emitted strong fluorescence signals [38]. Correspondingly, after the noise-exposed rat cochlea was treated with the antioxidant Q_10_ analog Q_ter_, the effect of noise exposure was offset, and the immune reaction was significantly reduced at the end of the treatment period [38]. Currently, the role of oxidative stress in NIHL and targeted therapy has not been systematically described. This article reviews the mechanism of oxidative stress in NIHL and the current therapeutic antioxidant drugs, aiming to clarify the prospects of targeting oxidative stress for NIHL treatment.

## Overview of the dysregulation of redox homeostasis: oxidative stress and reactive oxygen species (ROS)

### Oxidative metabolism: the basis of redox homeostasis

Reactive oxygen species (ROS) are composed of free radical oxygen and nonradical oxygen formed by the partial reduction of oxygen. ROS refer to various oxygen-derived compounds, free radicals and nonradicals, including superoxide anion O_2_^−^, hydrogen peroxide H_2_O_2_ and hydroxyl radical ·HO [40]. Intracellular ROS are produced endogenously during oxidative phosphorylation in mitochondria and can be produced via interactions involving exogenous substances [41]. Electron detachment during electron transport and binding to oxygen is the main source of ROS. Modifications of biomolecules mediated through low-level ROS are reversible switches of protein function and play an important role in cell proliferation, migration and differentiation [42]. However, excessive accumulation of ROS can be detrimental to the body's function.

**Fig. 1:**
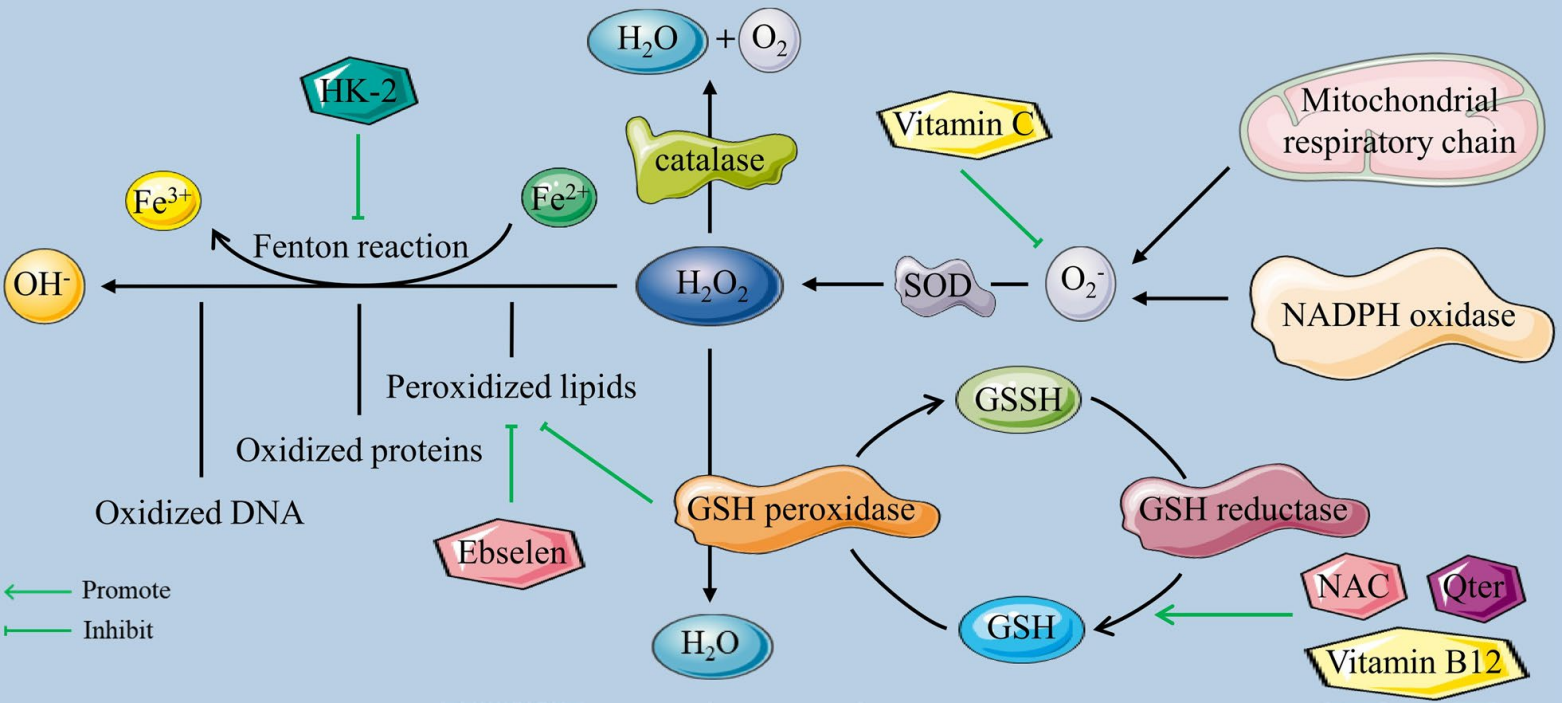
Major pathways of cellular ROS generation and detoxification

ROS are produced in many ways in cells, and the major pathways of cellular ROS generation and detoxification are shown in Fig. 1. Since selenocysteine(s) shows greater potential for oxidation than cysteine, the oxidative environment in the endoplasmic reticulum is supported by the expression of selenoproteins [40]. Ero1 proteins (flavoproteins) oxidize FADH2 to generate FAD and produce hydrogen peroxide with electrons and O_2_ [43]. Meanwhile, the protein-folding process in the endoplasmic reticulum produces immature oxidized glycoproteins, which subsequently enter the calnexin cycle [40].

The main function of NADP oxidase (NOX) is ROS production, which can affect many cellular components. The “ROS-dependent ROS generation” pathway is formed by the NOX-induced proliferation of ROS to generate other ROS-producing enzymatic systems [40]. NOXs are membrane proteins with multiple transmembrane regions, including binding sites for NADPH and FAD, and four histidine residues, which produce superoxide when generating molecular oxygen via heme [40].

In addition, the mitochondrial electron transport chain (ETC) generates highly active ·HO through a series of complex reactions, which indiscriminately oxidize proteins, lipids and DNA, resulting in DNA damage and genomic instability [44]. This critical process is described in detail in the next section.

Correspondingly, to maintain redox homeostasis, antioxidant systems in cells regulate ROS levels both temporally and spatially. Several antioxidants with enzymatic activity convert hydrogen peroxide into water, including peroxiredoxins (PRXs), catalase and glutathione peroxide (GPX) [4, 45]. Within mitochondria, NAD(P)H is the source of reducing equivalents for antioxidant GPX or PRX, and its oxidation is mediated via glutathione (GR) and thioredoxin (TR) reductase [46].

### Mitochondria: the main source of ROS

As the powerhouses of eukaryotic cells, mitochondria supply most of the energy in a cell by reducing oxygen in the ETC to produce ATP. However, the process of energy production via mitochondria is accompanied by ROS generation, which poses potential threats to mitochondria and physiological processes [47]. In the ETC, oxygen is reduced to water through the addition of two electrons. Although the efficiency of this process is very high, 1–4% of oxygen is not completely reduced through one-electron transfer. In fact, superoxide dismutase 2 (SOD2) in the mitochondrial matrix and SOD1 in the mitochondrial membrane gap convert O_2_^−^ to H_2_O_2_, which is converted to superoxide, the most common free radical. Then, superoxide diffuses into the cytoplasm through the mitochondrial membrane [40, 48]. Mitochondrial ROS (mtROS) are not only byproducts of the ETC; monoamine oxidase in the outer membrane and the α-ketoglutarate dehydrogenase complex are contributors to ROS production [49]. Low levels of mtROS enable metabolic adaptation and signal transduction functions. However, high levels of mtROS trigger inflammatory reactions via danger signaling and then activate autophagy and apoptosis [40]. When ROS are generated, antioxidants in the mitochondria maintain redox homeostasis under normal conditions. However, the oxidative stress generated by excessive ROS may lead to destructive consequences for mitochondria, other organelles, and cellular pathways, eventually leading to cell death [49].

## Oxidative stress-related mechanisms in NIHL

### Sources of ROS: destructive noise

The main sources of ROS in cells are mitochondria, and they have been thought to play a role in noise damage. On the one hand, extreme noise exposure directly causes intracellular rearrangements and mechanical damage to the cochlear structure, causing NIHL [50]. On the other hand, NIHL can be caused by more subtle forms of noise exposure [49]. Notably, ATP levels were depleted rapidly after noise exposure, indicating that mitochondria underwent metabolic changes after noise exposure [51]. Some researchers believe that the increase in the cochlear metabolic rate after noise exposure leads to excessive mitochondrial energy metabolism, which increases the rate of ROS leakage [52]. However, excessive ROS produced under stress situations show selective inhibition of the ETC, not its stimulation [53].

The effect of calcium homeostasis on ROS levels is widely recognized. ROS must pass through the mitochondrial membrane to enter the cytoplasm, and the destruction of mitochondrial membrane integrity and loss of membrane potential lead to the release of ROS into the cytoplasm [54]. Studies have shown that free Ca^2+^ in the cochlea increases immediately after excessive noise exposure [55], which may be a result of intracellular store release or entry via ion channels, including L-type or T-type voltage-gated calcium channels. Some experiments have proven that blocking these two types of voltage-gated calcium channels can attenuate the hearing loss caused by noise [56, 57], and extracellular Ca^2+^ can enhance the release of intracellular Ca^2+^, further increasing the level of free Ca^2+^ [58]. In addition, aminoglycoside antibiotics have been shown to alter the calcium homeostasis between the endoplasmic reticulum and mitochondria and thus promote the flow of Ca^2+^ from the endoplasmic reticulum to mitochondria [59, 60].

As another potential source of ROS, α-ketoglutarate dehydrogenase is a calcium-regulated Krebs cycle enzyme that can induce the generation of a large amount of superoxide and H_2_O_2_ [61]. In addition to increasing oxidative stress, dysfunctional α-ketoglutarate dehydrogenase increases mitochondrial free radical-induced damage and ETC inhibition, further increasing ROS production and initiating apoptotic pathways [61].

### Mechanisms of ROS damage to cochlear tissue

In the cochlea, different types of cells show different sensitivities to ROS. Because of different protein expression patterns, HCs seem to be the cochlear cells most vulnerable to damage caused by free radicals [62]. As stated above, ROS levels are increased in cochlear HCs exposed to excessive noise, either indirectly via NADPH oxidase and MAPK signaling cascades or directly via changes to cell morphology and the cell cycle, programmed death, and other physiological processes.

#### Peroxides, such as H_2_O_2_, directly damage HCs

H_2_O_2_, a typical ROS and a key signaling molecule, is suitable for studying the effects of oxidative stress on various physiological processes [63]. Through in vitro experiments based on mouse cochlear HCs, researchers have shown that a high H_2_O_2_ level increases the level of p53 protein, leading to apoptosis and necrosis. Moreover, the number of cells with nuclear blebbing and severely altered nuclear morphology was significantly increased, fewer of these cells were in the G1 phase of the cell cycle, and the number of these cells in the G2/M phase was increased, which may indicate delays in cell cycle progression [63]. In addition, H_2_O_2_ affects the antioxidant system, as indicated by the levels of GPX4, GSH and RSH in cells significantly increasing upon exposure to high levels of H_2_O_2_, which may lead to an imbalance in redox homeostasis and eventually trigger oxidative stress [63].

#### MAPK-mediated pathways: a transmitter of ROS damage

Mitogen-activated protein kinases (MAPKs) are involved in cellular signaling pathways and are important mediators of injury and survival signaling. MAPKs act downstream from plasma membrane receptors, intracellular receptors, and ROS [64, 65]. They activate gene expression via intermediate signaling proteins such as Src, Ras, Rac/cdc42, and mixed lineage kinase protein families. MAPKs are categorized into several different groups, among which c-Jun N-terminal kinases (JNKs) are clearly affected by ROS [66]. The MAPK family is comprised of the following three layers: MAPK, MAPK kinase (MAPKK) and MAPKK kinase (MAPKKK) [67]. MAPKKK phosphorylates and activates MAPKK; then, MAPKK phosphorylates and activates MAPK, which phosphorylates the target substrate [68]. Intense noise has been proven to affect the phosphorylation of MAPK in the cochlea and lead to HC death [69–71]. In addition, enrichment analysis of the KEGG pathway confirmed that the MAPK signaling pathway was significantly enriched pathway of differentially expressed proteins in the inner ear of NIHL rats [72].

JNK is expressed as three isomers, JNK1, JNK2 and JNK3, among which JNK1 and JNK2 are extensively expressed, and JNK3 is expressed mainly in heart and nerve tissues. When exposed to extracellular stimulation, JNK signaling can reach the nucleus and mitochondria, promoting apoptosis via either pathway [66]. Specifically, a JNK signal that is transported to the nucleus phosphorylates c-Jun and other transcription factors, including p53, and then promotes the expression of proapoptotic genes (FasL, TNF α, Bim, Bak, etc.), which blocks the transcription of antiapoptotic genes (such as Bcl-2) [73–80]. In the other pathway, activated JNK can phosphorylate a variety of proteins related to mitochondrial cell death. Phosphorylated JNK indirectly triggers proapoptotic Bax-mediated mitochondrial death signaling through the cleavage of Bid. In this regard, studies have confirmed that Bax expression is upregulated in noise-exposed rats [81]. Additionally, after phosphorylation by activated JNK, the death-promoting proteins Bim and Bad stop the inhibition of the Bcl-2 and Bcl-xL proteins and promote Bax activation of the intrinsic apoptosis pathway [82–87]. The increase in ROS levels promotes the prolongation of JNK activation, which in turn leads to an increase in mitochondrial ROS production, forming a positive feedback loop [66, 88]. Correspondingly, JNK inhibitors have been proven to exert a protective effect on HC damage caused by noise exposure [89–91], which verifies the accuracy of the description of the aforementioned mechanisms.

## Antioxidant treatment of NIHL

Biological antioxidants can delay or prevent the oxidation of substrates when the concentration is lower than that of the oxidizable substrate [92], inhibiting the activity of ROS and reducing oxidative stress, DNA mutation, malignant transformation and other cell-damaging events. However, this steady state may be altered under the action of sustained free radicals, leading to disease [92, 93].

The research shows that the peroxidation reaction starts from the addition of oxygen to carbon-centered radicals, with most of the hydrogen atoms transferred to the chain carrying peroxyl radicals. Antioxidants reduce the local concentration of molecular oxygen, remove pro-oxidative metal ions, capture aggressive ROS (superoxide anion radicals and hydrogen peroxide, etc.), remove chain-starting free radicals (hydroxyl, peroxyl, alkoxyl, etc.), inhibit the sequential production of radical chains, and quench singlet oxygen [94, 95]. The redox homeostasis of cells is maintained by complex endogenous antioxidant defense systems, including endogenous antioxidant enzymes (catalase, superoxide dismutase, glutathione peroxidase, etc.) and nonenzymatic compounds (glutathione, metal-chelating proteins, etc.) [96]. In addition, exogenous antioxidants are capable of synergistically reducing oxidative stress and impacting related disease processes [96, 97]. Hence, based on these mechanisms, antioxidants and their analogs have become the main components in antioxidant treatment strategies. There have been many drug studies targeting NIHL based on these findings, and we will discuss some potential drugs.

### Activation of endogenous antioxidants: the core antioxidant system

The Kelch‐like ECH‐associated protein 1 (Keap1)–nuclear factor erythroid 2‐related factor 2 (Nrf2)–antioxidant response element (ARE) pathway is the center of the biological response to oxidative stress, regulating a variety of cell-protecting proteins (also known as phase 2 enzymes) to maintain the homeostasis of ROS levels in cells [98–102]. Under oxidative stress conditions, the Nrf2 protein is stabilized and transferred to the nucleus, where it activates a variety of ARE-response genes, including antioxidant genes, heme oxygenase-1 (HO-1) and nicotinamide adenine dinucleotide phosphate (NAD(P)H) [101–104]. The Nrf2 pathway is also activated by the interruption of the interaction between Nrf2 and Keap1 [105]. p62 is a ubiquitin-binding protein that activates Nrf2 in autophagy-deficient cells by disrupting the Keap1-Nrf2 interaction [106]. p62 is upregulated under oxidative stress, leading to the sequestration of Keap1 and activating Nrf2 and Nrf2-dependent antioxidant defense gene expression [107]. The Nrf2-ARE pathway targets a wide range of downstream proteins, which are listed in Table 1. In this section, we describe in detail several major endogenous antioxidant enzymes that have been studied and shown to exert effects in NIHL.

**Table 1:** Target proteins in the Nrf2-ARE pathway

Target proteins	Results	Mechanism	References
GSH synthetic enzymes↑ GSH regenerative enzymes↑	GSH↑	Increases GSH synthesis and regeneration, improves GSH levels	Stefanson et al. [113]
Glutamate cysteine ligase (GCL)↑	GSH↑	Upregulates two subunits of GCL, GCLC and GCLM, catalyzes the rate-limiting step in GSH synthesis	Shelly et al. [114]
HO-1↑	Heme↓	The inducible isoform of HO-1 removes the oxidation promoting molecule heme	Maines et al. [109]
	Bilirubin↑ Biliverdin↑	HO-1 decomposes heme to produce biliverdin and its reduction product bilirubin, both of which have antioxidant effects	Clark et al. [110]
NAD(P)H generating enzyme	NAD(P)H	Keap1 increases mitochondrial NADH pool	Holmström et al. [119]
		Nrf2 expression of aldehyde dehydrogenase, generates NAD(P)H with NAD(P) as a cofactor	Dinkova-Kostova et al. [102]
Zn/Cu-SOD↑	Superoxide anion radicals↓	Nrf2 increases the level of Zn/Cu-SODs, Zn/Cu-SODs catalyze the disproportionation of superoxide anion radicals to generate oxygen and hydrogen peroxide	Tu et al. [105]

The symbol ↓ means the target factor level is reduced, and the symbol ↑ means the target factor level rises

Fluorescence analysis showed that Nrf2, HO-1 and SOD levels were slightly increased in cells damaged by noise, revealing an adaptive stress response. In the same HC and SGN samples used for the fluorescence assay, Nrf2 translocation paralleled the increase in expression of HO-1 [108]. This means that Nrf2-induced upregulation of HO-1 affects OHC signal amplification and transmission from primary afferent neurons to the central acoustic pathway [4]. The inducible isoform of HO-1 plays an antioxidative role by removing the oxidation-promoting molecule haem, thereby acting as a microsomal enzyme in haem catabolism [109]. The catabolic products of haem include biliverdin, which is converted into bilirubin after reduction via biliverdin reductase. Both haem and biliverdin exert significant antioxidant effects, contribute to resistance to oxidative stress, and protect cells [109–111]. Iron is released during haem catabolism, which leads to the upregulation of ferritin. As an iron-storing protein, ferritin participates in HO-1 activity against oxidative stress [111, 112].

In addition, an increase in GSH production and the activity of its scavenger has been shown to be induced by Nrf2 [108, 113, 114]. Glutathione is the most abundant antioxidant and plays a major role in oxidative stress resistance [115]. When Nrf2 and HO-1 levels are increased, the amount of GSH also increases. Nrf2 not only regulates GSH synthesis but also coordinates GSH reductase transcription. Nrf2 leverages the reducing equivalents of NAD(P)H to reduce oxidized GSH, thus maintaining it in the reduced state [116–118].

Furthermore, Nrf2 regulates the major NAD(P)H-generating enzyme, aldehyde dehydrogenase, via transcription, inducing aldehyde dehydrogenase expression in microsomes, the cytoplasm and mitochondria and generating NAD(P)H with the cofactor NAD(P) [102]. Compared with that in Keap1-wild-type cells, the total mitochondrial NADH pool in Keap1-KO cells was significantly increased, and it was significantly decreased in Nrf2-KO cells [119]. Researchers analyzed the NAD(P)H reduction capacity of OHCs and found that after noise exposure, the oxidation of NAD(P)H increased, the plasma membrane peroxidation rate increased, and plasma membrane fluidity decreased concomitantly [120–122]. The accumulation of free radicals leads to plasma membrane peroxidation and then triggers lipid peroxidation, indicating that effective antioxidant treatment may enhance the protection of OHCs from cell death [4].

### Exogenous antioxidants: prospects for drug intervention

Endogenous antioxidants play an extremely important role in maintaining redox homeostasis. When the concentration of endogenous antioxidants is insufficient to maintain redox homeostasis, exogenous antioxidants are considered effective for inducing oxidative stress resistance. On this basis, antioxidants that regulate cellular oxidative stress to treat NIHL by targeting free radicals have been developed [123]; some of these antioxidants are listed in Table 2. In the remainder of this section, we describe several drugs that have been extensively studied.

**Table 2:** Potential antioxidants for noise-induced hearing loss

Antioxidants	Mechanisms	Experimental results	Animal models	References
N-acetylcysteine (NAC)	Provides substrate for GSH synthesis, inhibits the activation of MAPKs pathway, and scavenges free radicals	Effectively attenuates lipid peroxidation in the cochlea of guinea pigs and provides significant protection from hair cell and hearing loss	Noise-exposed guinea pig model (5 h)	Kopke et al. [127]
Ebselen	Mimics and enhances the activity of GPX1, significantly inhibits ROS and lipid peroxidation, increases the transcriptional activity of ARE and the expression level of HO-1 protein	Significantly reduces ABR threshold shift and loss of OHC in rats	Noise-exposed rat model (4 h)	Alvarado et al. [123] Kim et al. [137]
Vitamin A	Removes singlet oxygen, prevents lipid peroxidation	Significantly reduces the ABR threshold and protects HCs survival in guinea pigs	Noise-exposed guinea pig model (5 h)	Le Prell et al. [141]
Vitamin C	Scavenges oxygen free radicals in the aqueous phase, blocks and/or reverts lipid peroxidation in the plasma membrane, regenerates vitamin E from the oxidized form	Significantly reduced PTS and OHCs damage in guinea pigs	Noise-exposed guinea pig model (6 h)	McFadden et al. [148]
Vitamin E	Inhibits the proliferation cycle of lipid peroxidation by reacting with peroxyl radicals and reducing their quantity	Significantly reduces the ABR threshold and protects HCs survival in guinea pigs	Noise-exposed guinea pig model (5 h)	Le Prell et al. [141]
Vitamin B12	Decreases homocysteine levels, leading to increased intracellular glutathione concentrations and inhibiting lipid peroxidation	Surveys based on NIHL patients showed B12 have a significant protective effect on NIHL	Army personnel exposed to military noise	Gok et al. [151] Shemesh et al. [150]
HK-2	reduces the oxidative stress generated by free radicals, synthesizes bioactive transition metals including Fe^2+^, thus reducing their availability to participate in the Fenton reaction that produces highly toxic hydroxyl radicals	reduces noise-induced hearing impairment (reflected in cochlear compound action potential) and hair cell loss in rats	Noise-exposed rat model (8 h/d for 21 days)	Chen et al. [156]
Qter	As the artificial analog of an endogenous antioxidant coenzyme Q10, involves in free radicals scavenging and regeneration of antioxidants like reduced GSH	Promotes outer hair cell (OHC) survival in a guinea pig NIHL model	Noise-exposed guinea pig model (60 min)	Fetoni et al. [38]
Folic Acid	Promotes homocysteine metabolism, decreases superoxide metabolism	Folic acid-deficient mice exhibit impaired cochlear homocysteine metabolism and associated oxidative stress	C57BL/6 J female mice (Due to the Ahl alleles present in the C57BL/6 J mouse strain’s genome, these mice demonstrate ARHL from the age of 6 mo onward)	Martínez-Vega et al. [152]
Acetyl-L carnitine (ALCAR)	Serves as a precursor for L-carnitine, which can shuttle lipid substrates into mitochondria for β-oxidation and enhance ATP production, restores cardiolipin in oxidatively injured cells, further restoring mitochondrial integrity	Significantly reduces hearing threshold shift and loss of OHCs in chinchilla lanigers	Noise-exposed chinchilla laniger model (6 h)	Kopke et al. [39]

#### N-Acetylcysteine (NAC)

A precursor of cysteine, NAC is a substrate antioxidant in glutathione synthesis [124–126] that may inhibit oxidative stress and reduce cochlear damage caused by noise exposure by providing a substrate for GSH synthesis, inhibiting the activation of the MAPK pathway, and scavenging free radicals [125, 127]. As a drug approved by FAD, oral NAC has been used clinically for more than 30 years, even in very large doses (8 g per day for 8 weeks), and it causes only mild side effects [125, 128]. However, as the synthesis of glutathione decreases with age, the effectiveness of NAC decreases with age [126]. Since ROS are considered necessary for physiological cellular activities, a certain ROS level must be maintained for drugs such as NAC to be used as an effective treatment [41].

Performing animal experiments, researchers found that NAC minimized the apoptosis rate of sensory HCs and significantly reduced low- and medium-frequency auditory threshold shifts [124, 129]. When used in humans, oral NAC is metabolized into cysteine in the intestine, and this cysteine is oxidized to cystine, which is then transported into cells to be consumed as an essential precursor for glutathione synthesis [125]. In fact, the effect of the cysteine product is more significant than that of NAC treatment. However, due to differences in intestinal and visceral metabolism, the bioavailability of cysteine after oral administration of NAC is very high, which makes NAC a better supplement than cysteine for consumption as a GSH precursor [126, 130, 131].

In addition, NAC has been shown to inhibit JNK, p38 MAP kinase and NF-κB pathway activation, and the inhibition of these signaling pathways reduces the corresponding cell damage and apoptosis rate [132, 133]. Moreover, NAC has also been found to be a mitochondrial protectant in different models of stress induction [134, 135]. Therefore, NAC is thought to attenuate NIHL via multiple antioxidant mechanisms.

#### Ebselen

Ebselen is a selenium-based organic compound with antioxidant properties that can mimic and enhance the activity of GPX1 [123, 136]. Immunofluorescence assays of rat cochlea showed that the weighted intensity of the GPX1-expressing area in the rat cochlea decreased by 8.8% after noise exposure compared with a decrease in the no-noise controls, but the weighted intensity of the GPX1-expressing area in the noise-exposed rat cochlea after ebselen treatment increased by 9.9% compared with the level in the no-noise controls [136]. This study shows that ebselen plays a role in enhancing GPX1 expression.

In fact, ebselen shows multifunctional ear protection activity. In other experiments, ebselen was found to exert a significant inhibitory effect on ROS production and lipid peroxidation [137]. Additionally, ebselen enhanced the activity of the cellular antioxidant system. At a certain concentration, ebselen significantly increased the transcriptional activity of ARE and the expression level of the HO-1 protein, contributing to the fight against oxidative stress [137].

Previous studies have shown that the immediate injection of ebselen into rats before and after NIHL induction can effectively reduce auditory thresholds and HC death [136]. The safety and limited but significant efficacy of ebselen in humans have also been proven, providing a basis for the future use of ebselen in clinical treatment [138].

#### Vitamins

Some micronutrients, such as vitamins A, C, E and B12, exhibit antioxidant properties. They are considered to exert synergistic effects and have been used either individually or in combination for the experimental treatment of NIHL.

The main antioxidant effect of vitamin A is the removal of singlet oxygen. Since singlet oxygen and lipids are substrates of lipid peroxide, removing singlet oxygen can prevent lipid peroxidation [139–143]. When retinoic acid (the most active metabolite of vitamin A) was injected into noise-exposed mice, the apoptosis rate of HCs was significantly reduced, the cochlea was protected, and the auditory threshold was also restored rapidly [144–146].

Vitamin C is a water-soluble molecule; therefore, the elimination of oxygen radicals by vitamin C occurs in the aqueous phase and is very effective in blocking and/or reverting lipid peroxidation at the plasma membrane [147, 148]. Studies have shown that taking vitamin C before excessive noise exposure significantly reduced the TTS in rats [149]. Furthermore, vitamin C protects cell membranes by regenerating vitamin E from its oxidized form, and vitamins C and E play opposite roles, which shows that the oxidation of vitamin C and E is coupled [141, 143].

The antioxidation mechanism of vitamin E is closely related to vitamin C. Vitamin E exists in lipids in cells and is a main free radical scavenger of cell membranes. It inhibits the proliferation cycle of lipid peroxidation by reacting with peroxyl radicals and reducing their number [140–143]. Researchers gave vitamins A, C, and E and magnesium to animals exposed to noise and found that the threshold of the audit brain response (ABR) in the animals treated with combined administration was significantly reduced, and the HCs of these animals survived [141].

Vitamin B12 has been found to be associated with NIHL. Researchers found that the incidence of vitamin B12 deficiency in NIHL patients was higher than that in subjects with normal hearing. Further studies showed that the serum vitamin B12 and folic acid levels in NIHL patients were significantly lower than those in subjects without NIHL [150–152]. Deficiency in vitamin B12 and folic acid increases the level of homocysteine, which may lead to a decrease in the intracellular glutathione concentration, thus increasing lipid peroxidation [151, 153].

Comprehensive research has shown that the combined treatment with vitamins A, C, and E and magnesium taken one hour before noise exposure significantly reduced NIHL and cell death, and long-term high-dose intake of these micronutrients showed no obvious adverse effects [141, 153]. These studies provide a direction and basis for the use of vitamins in the treatment of NIHL.

#### HK-2

HK-2 is a multifunctional antioxidant with the characteristics of a free radical scavenger and metal chelator [154–156]. Therefore, in addition to reducing the oxidative stress generated by free radicals, HK-2 synthesizes bioactive transition metals, including Fe^2+^, thus reducing the availability of these ions for Fenton reactions, which produce highly toxic hydroxyl radicals [156].

In an in vitro oxidative stress experiment, HK-2 showed a more significant protective effect than Trolox in reducing hydrogen peroxide damage, hydroxyl damage and peroxynitrite damage. With respect to superoxide damage, both Trolox and HK-2 were effective in inhibiting mitochondrial superoxide production in rat cochlear cells [156]. This finding suggests that HK-2 significantly inhibits damage caused by a variety of NIHL-related peroxides [157, 158].

Taking HK-2 before noise exposure can have a significant and particularly substantial protective effect. A dose-dependent reduction in hearing threshold shifts was obtained in rats after continuous treatment with HK-2 for 5 days prior to noise exposure, and this protection lasted for 10 days postexposure [156]. In addition, preventive treatment with HK-2 significantly reduced the pathological changes in OHCs. Notably, the magnitude of the protective effect of HK-2 orally ingested 10 days after exposure was profoundly lower than that of the preventive treatment [156].

In conclusion, HK-2 has been shown to significantly reduce NIHL, attenuate HC loss in rats and significantly reduce ROS-induced cell loss and damage. Furthermore, HK-2 did not show any adverse effects in rats [154–156], suggesting that HK-2 is a new therapeutic option for NIHL.

## Conclusions and perspectives

Oxidative stress plays a pivotal role in many diseases, and its complex and diverse mechanisms indicate a variety of targets for possible treatments. In contrast, therapeutic options for NIHL, a disease with a wide impact, are rare. Oxidative stress-related pathways have been found to be significantly variable and specific in NIHL. Therefore, in-depth study of oxidative stress and NIHL is warranted. In this paper, we provide a review of the mechanisms of NIHL and oxidative stress and describe analyses that link pathways in NIHL and oxidative stress, establishing a starting point to develop new and promising drug treatments.

Antioxidants are promising therapeutic modalities for NIHL because they can counteract oxidative stress directly or indirectly. A variety of antioxidants have been shown to protect animal HCs from damage, thereby reducing hearing threshold shifts and attenuating NIHL [159–162], and because noise exposure is often predictable, prophylactic treatment with NIHL is a highly feasible option that has been tested with varying degrees of success. Prophylactic treatment allows for the utilization of many micronutrients that are usually ingested daily and induce very few side effects [143], providing a significant advancement in NIHL treatment options.

However, because of the difficulty of performing biopsies of cochlear tissue, drug trials for NIHL have been limited to animal models, and these experiments or direct observations are difficult to translate to humans. Therefore, antioxidants that have been validated in animal studies still need to be subjected to follow-up experiments to determine their side effects in humans when administered alone or in combination. While these problems have limited the research on oxidative stress-related drugs, the progress to date portends great potential for these treatments.

## Data Availability

All data generated or analyzed during this study are included in this published article and references.

## Abbreviations

ROS: Reactive oxygen species
NIHL: Noise-induced hearing loss
HC: Hair cell
TTS: Temporary hearing threshold shift
ANF: Auditory nerve fiber
PTS: Permanent threshold shift
OHC: Outer hair cell
SGN: Spiral ganglion neuron
DAMP: Damage-associated molecular pattern
TLR: Toll-like receptor
RAGE: Receptor for advanced glycation end product
NOX: NADP oxidase
ETC: Electron transport chain
PRX: Peroxiredoxin
GPX: Glutathione peroxide
SOD2: Superoxide dismutase 2
mtROS: Mitochondrial ROS
MAPK: Mitogen-activated protein kinase
JNK: C-Jun N-terminal kinase
MAPKK: MAPK kinase
MAPKKK: MAPKK kinase
Keap1: Kelch‐like ECH‐associated protein 1
Nrf2: Nuclear factor erythroid 2‐related factor 2
ARE: Antioxidant response element
HO-1: Haem oxygenase-1
NAD(P)H: Nicotinamide adenine dinucleotide phosphate
ABR: Audit brain response
